# Annotated genome sequences of *Salmonella* Haifa, *Salmonella* Bangkok, and *Salmonella* Reading, isolated from chicken meat in South Africa

**DOI:** 10.1128/mra.00284-24

**Published:** 2024-10-09

**Authors:** Masenyabu Mathole, Laura Carroll, Collette Khabo-Mmekoa, Nomsa Mabogoane, Itumeleng Matle

**Affiliations:** 1 Bacteriology Laboratory, Agricultural Research Council-Onderstepoort Veterinary Research, Onderstepoort, South Africa; 2 Department of Biomedical Sciences, Tshwane University of Technology, Pretoria, South Africa; 3 Department of Clinical Microbiology, SciLifeLab, Umeå University, Umeå, Sweden; 4 Laboratory for Molecular Infection Medicine Sweden (MIMS), Umeå University, Umeå, Sweden; 5 Umeå Centre for Microbial Research, Umeå University, Umeå, Sweden; 6 Integrated Science Lab, Umeå University, Umeå, Sweden; University of Maryland School of Medicine, Baltimore, Maryland, USA

**Keywords:** *Salmonella* Haifa, *Salmonella* Bangkok, *Salmonella* Reading, annotated, genomes, chicken meat, South Africa

## Abstract

This paper presents the annotated genomes of *Salmonella* Haifa, *Salmonella* Bangkok, and *Salmonella* Reading, which are uncommonly isolated from meat in South Africa. Despite their rarity in South Africa, these serotypes have been linked to several high-profile outbreaks in other parts of the world.

## ANNOUNCEMENT

*Salmonella* Haifa, *Salmonella* Bangkok, and *Salmonella* Reading are uncommon in South Africa but have recently been isolated from various sources (1). The latter is rarely isolated but associated with sporadic cases and outbreaks of human salmonellosis worldwide (2). A multistate salmonellosis outbreak in the USA caused by *S*. Reading from contaminated turkey meat was reported (3). *S*. Haifa was implicated in a fatal food poisoning case of a 78-year-old man in Japan (4). There is limited information available for *S*. Bangkok, including cases and virulence characteristics. The lack of understanding about these emerging serovars and their potential threat to animals and humans in South Africa is concerning.

Here, we announce the genome sequences of *S*. Haifa SRR28112779, *S*. Bangkok SRR28112783, and *S*. Reading SRR28112782. The isolation and identification were performed using microbiological methods according to ISO 6579-1: 2017 (5). The strains were isolated from chicken meat.

DNA was isolated from overnight cultures incubated at 35°C ± 1°C on blood agar plates using High Pure Template Preparation Kit (Roche, Johannesburg, South Africa). DNA libraries were prepared using the TruSeq DNA Library Preparation Kit (Illumina, Inc., San Diego, CA, USA). The resulting library was sequenced on Illumina HiSeq 2500 (Illumina, San Diego, CA, USA), generating 150-bp paired-end reads; reads were trimmed using Trimmomatic version 0.39 in PE mode, using the adapter file provided by Shovill version 1.1.0 and the following parameters: ILLUMINACLIP:trimmomatic.fa:1:30:11 LEADING:3 TRAILING:3 MINLEN:30 TOPHRED33 (https://github.com/tseemann/shovill/). Read quality was evaluated using FastQC version 0.12.1 (default settings; https://www.bioinformatics.babraham.ac.uk/projects/fastqc/) () and MultiQC version 1.16 (default settings) (6), resulting in a total of (i) 4,536,288 for SRR28112779, (ii) 4,851,551 for SRR28112783, and (iii) 4,824,872 for SRR28112782. Genomes were assembled using Shovill version 1.1.0 and the following parameters: (i) SKESA version 2.4.0 as assembler, (ii) a minimum coverage of 10, and (iii) a minimum contig length of 200 bp (7). Genome quality was evaluated using QUAST version 5.2.0 (default settings, except “--min-contig 1”) (8) and CheckM version 1.2.2 (default settings with “lineage_wf” workflow) (9). The “classify_wf” workflow in GTDB-Tk version 2.3.2 (10) was used to confirm that all genomes belonged to *Salmonella enterica* (using default settings and release214 of the Genome Taxonomy Database), and mlst version 2.9 (https://github.com/tseemann/mlst) was used to assign each genome to an ST via *in silico* seven-gene MLST (default settings). Genomes were serotyped *in silico* using (i) SISTR version 1.1.1 (with “--qc” option) (11) and (ii) SeqSero2 version 1.2.1 (in assembly and *k*-mer mode with “-t 4” and “-m k”) (12). Genomes were submitted to NCBI’s GenBank database on 27 February 2024 (https://www.ncbi.nlm.nih.gov/) and annotated using NCBI’s Prokaryotic Genome Annotation Pipeline (13).

SRR28112779, SRR281127783, and SRR28112782 genomes were 4,761,512, 4,695,697, and 4,566,845 bp in length with GC contents of 52.27%, 52.12%, and 52.17%, respectively. Genomes were further composed of 46, 119, and 29 contigs and had *N*_50_ values of 376,927, 141,264, and 398,030 bp, and average genome coverage was 284, 304, and 315, respectively. The genomes’ sequence types and number of predicted genes were ST2656 and 4,667 for SRR28112783, ST412 and 4,461 for SRR28112782, and no ST and 4,688 for SRR28112779.

## Data Availability

The whole-genome assembly sequences were deposited in the NCBI database under BioProject accession number PRJNA1081203. The SRA accession numbers are SRR28112779, SRR28112783, and SRR28112782 for Illumina reads.
